# Supplementary material for: Mechanical on-chip microwave circulator
Source: Nat Commun. 2017 Oct 16;8:953. doi: 10.1038/s41467-017-01304-x (PMC5643437; doi:10.1038/s41467-017-01304-x)
Supplement: Supplementary file 1 — Supplementary Information [file 41467_2017_1304_MOESM1_ESM.pdf]

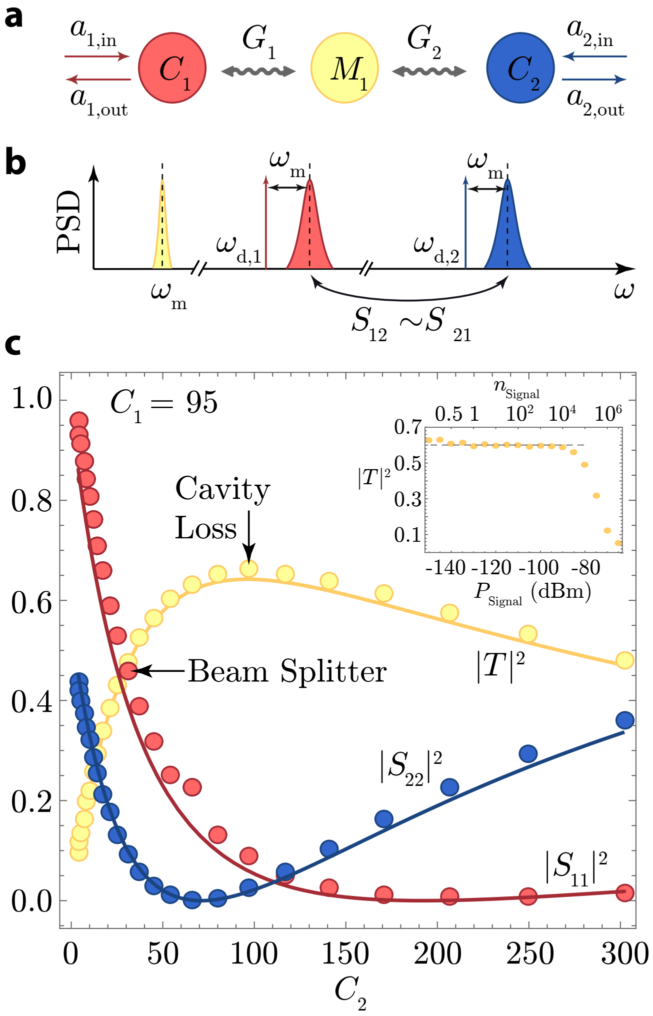

Supplementary Figure 1. **Bidirectional frequency conversion a**, The microwave-mechanical mode diagram for the frequency conversion. Two microwave cavities C_1 and C_2 are parametrically coupled to a mechanical mode with coupling rates G_1 and G_2, which gives rise to frequency conversion between the two microwave cavities. **b**, Power spectral densities (PSD) of the mechanical mode and microwave cavities and the drive tone frequencies indicated with vertical arrows near the red sidebands of the microwave modes at $\omega_{\text{d},1(2)} = \omega_{1(2)} - \omega_\text{m}$. **c**, Experimental demonstration (dots) and theoretical prediction (solid lines) of the frequency conversion between two microwave cavities at resonance frequencies $(\omega_1, \omega_2)/2\pi = (9.55, 9.82)$ GHz as a function of cooperativity C_2 for $C_1 = 95$. Here, $|T|^2 = |S_{12}| \cdot |S_{21}|$ (yellow dots), $|S_{11}|^2$ (red dots) and $|S_{22}|^2$ (blue dots) demonstrate the magnitude of the transmission and reflection coefficients on resonance with the cavities, respectively. As predicted by Eq. (3), the transmission between the two cavities is maximum for $C_1 = C_2 \approx 95$. The inset shows the dynamic range of the device where the transmission coefficient is measured as function of the signal input power P_signal or mean total number of signal photons inside the microwave cavities n_signal.

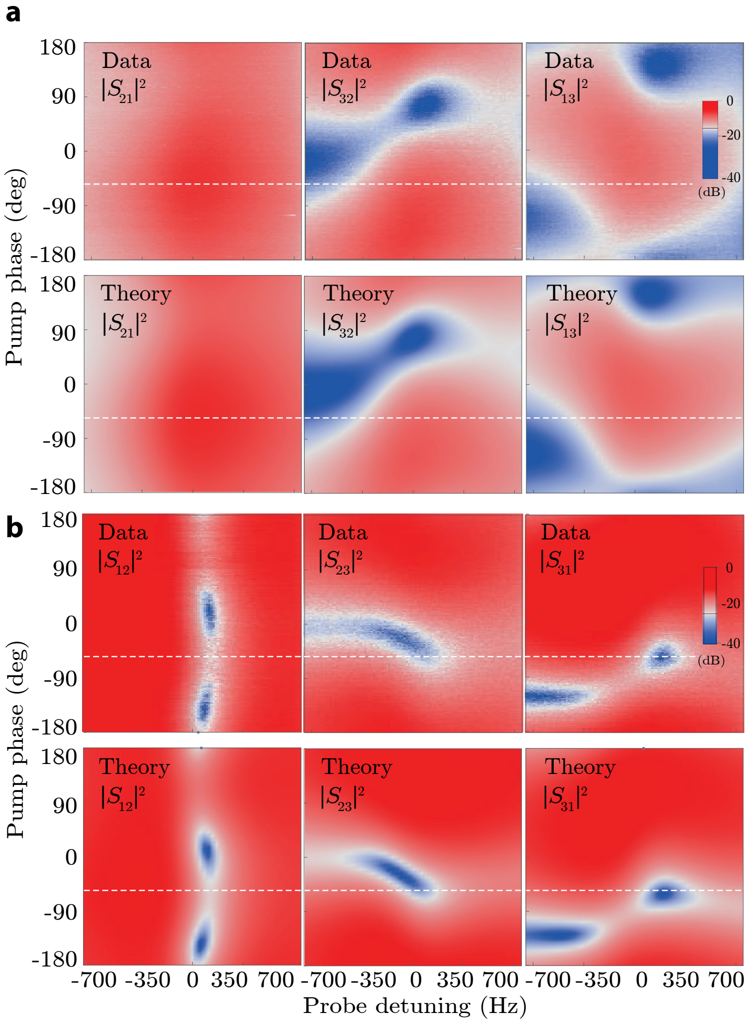

Supplementary Figure 2. **Full scattering parameters of the circulator.** **a**, Measured power transmission and theoretical model in forward direction ($|S_{21}|^2$, $|S_{32}|^2$, and $|S_{13}|^2$) as a function of detuning and pump phase. **b**, Measured power transmission and theoretical model in backward direction ($|S_{12}|^2$, $|S_{23}|^2$, and $|S_{31}|^2$) as a function of detuning and pump phase.

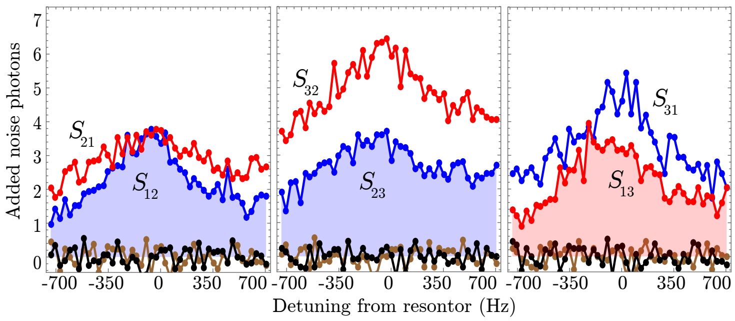

Supplementary Figure 3. **Added noise photons of the circulator.** Measured circulator noise properties in forward direction ($|S_{21}|^2$, $|S_{32}|^2$, and $|S_{13}|^2$ in red) and backward direction ($|S_{12}|^2$, $|S_{23}|^2$, and $|S_{31}|^2$ in blue) as well as the measured background noise (forward, black symbols and backward, brown symbols) as a function of detuning.

	$\frac{\omega}{2\pi}$(GHz)	$\frac{\kappa_{\rm int}}{2\pi}$(MHz)	$\frac{\kappa_{\rm ex}}{2\pi}$(MHz)	$\frac{\kappa}{2\pi}$ (MHz)	$\eta = \frac{\kappa_{\rm ex}}{\kappa}$	L(nH)	$C_{\rm s}$(fF)	$C_{\rm m}$(fF)	$\frac{g_{01}}{2\pi}$(Hz)	$\frac{g_{02}}{2\pi}$(Hz)
Cavity 1	9.55	0.62	1.8	2.42	0.74	48.2	5.3	0.45	33	34
Cavity 2	9.82	0.28	1.7	1.98	0.86	48.3	4.98	0.45	13	31
Cavity 3	11.32	1.42	1.58	3	0.52	34.4	5.29	0.45	22	45

Supplementary Table 1. Microwave resonator properties.

	$\frac{\omega_{\rm m}}{2\pi}$(MHz)	$\frac{\gamma_{\rm m}}{2\pi}$(Hz)	$m_{\rm eff}$(pg)	$x_{\rm zpf}$ (fm)
first mechanical mode	4.34	4	4	22
second mechanical mode	5.64	8	2.2	26

Supplementary Table 2. Mechanical properties.

Supplementary Note 1: Circuit properties

The electromechanical microwave circuit shown in Fig. 1 a of the main text, includes three high-impedance microwave spiral inductors (L_i) capacitively coupled to the in-plane vibrational modes of a dielectric nanostring mechanical resonator, creating three LC resonators with frequencies $\omega_i = 1/\sqrt{L_i C_i}$ with $i = 1, 2, 3$. The nanostring resonator fabricated from a high resistivity smart-cut silicon-on-insulator wafer with 220 nm device layer thickness has a length of 9.4 µm and consists of two metallized beams that are connected with two tethers at their ends. The vacuum gap size for the mechanically compliant capacitor fabricated with an inverse shadow technique [1] is approximately 60 nm.

The electromechanical coupling between the nanostring mechanical resonator and each LC circuit is given by

$$g_{0i} = x_{\rm zpf}\frac{\partial\omega_i}{\partial v} = -x_{\rm zpf}\zeta_i\frac{\omega_i}{2C_{{\rm m},i}}\frac{\partial C_{{\rm m},i}}{\partial v}, \tag{1}$$

where v is the amplitude coordinate of the in-plane mode, $\zeta_i = \frac{C_{{\rm m},i}}{C_{\Sigma,i}}$ is the participation ratio of the vacuum gap capacitance $C_{{\rm m},i}$ to the total capacitance of the circuit $C_{\Sigma,i} = C_{{\rm m},i} + C_{{\rm s},i}$, where $C_{{\rm s},i}$ is the stray capacitance of the circuit including the intrinsic self-capacitance of the inductor coils. Eq. (1) indicates that large electromechanical coupling g_{0i} requires a large participation ratio. We can make the coil capacitance $C_{{\rm L},i}$ relatively small by using a suspended and tightly wound rectangular spiral inductor with a wire width of 500 nm and wire-to-wire pitch of 1 µm [2]. Knowing the inductances L_i of the fabricated inductors based on modified Wheeler, as well as the actually measured resonance frequencies ω_i along with vacuum-gap capacitance $C_{\rm m}$ (from FEM simulations), we can find the total stray capacitance including the intrinsic self-capacitance of the each inductor coil correspondingly. Careful thermometry calibrated mechanical noise spectroscopy measurements similar to the ones in [2] yield the measured electromechanical coupling for each mode combination as outlined in the Supplementary Table 1.

We use finite-element method (FEM) numerical simulations to find the relevant in-plane mechanical modes of the structure and optimize their zero point displacement amplitudes and mechanical quality factor. Our simulations are consistent with the measured mechanical frequencies for a tensile stress of ∼600 MPa in a ∼70 nm thick electron beam evaporated aluminum layer [3]. The associated effective mass and zero-point displacement amplitude along with the measured linewidths and resonance frequencies of the first two in-plane modes of the nanostring are presented in the Supplementary Table 2.

Supplementary Note 2: Bidirectional frequency conversion

To understand the electromechanical frequency conversion, we first theoretically model our system to see how frequency conversion arises. Supplementary Figure 1 a shows an electromechanical system, in which two microwave cavities with resonance frequencies ω_1 and ω_2 and linewidths κ_1 and κ_2 are coupled to a mechanical oscillator with frequency $\omega_{\rm m}$ and damping rate γ. The electromechanical coupling is driven by two strong drive fields, $\mathcal{E}_1$ and $\mathcal{E}_2$, near the red sideband of the respective microwave modes at $\omega_{{\rm d},1(2)} = \omega_{1(2)} - \omega_{\rm m}$, see Supplementary Fig. 1 b. In the resolved-sideband limit ($\omega_{\rm m} \gg \kappa_{1(2)}, \gamma$) the linearized electromechanical Hamiltonian in the rotating frames with

respect to the external driving fields is given by ($\hbar = 1$)

$$H = \sum_{i=1,2} \Delta_i a_i^\dagger a_i + \omega_\mathrm{m} b^\dagger b + \sum_{i=1,2} G_i \Big(a_i b^\dagger + b a_i^\dagger \Big), \tag{2}$$

where $a_{1(2)}$ is the annihilation operator for the microwave signal field 1 (microwave signal field 2), b is the annihilation operator of the mechanical mode, $\Delta_{1(2)} = \omega_{1(2)} - \omega_{\mathrm{d}1(2)} = \omega_\mathrm{m}$ is the detuning between the external driving field and the relevant cavity resonance, and $G_i = g_{0i}\sqrt{n_i}$ is the effective electromechanical coupling rate between the mechanical resonator and cavity i with $n_i = \frac{2\mathcal{E}_i}{\kappa_i^2 + 4\Delta_i^2}$ being the total number of photons inside the cavity. Note that, the fast-oscillating counter-rotating terms at $\pm 2\omega_\mathrm{m}$ are omitted from the Hamiltonian under the rotating wave approximation.

The first and second terms of Hamiltonian (2) describe the free energy of the mechanical and cavity modes while the last term of the Hamiltonian indicates a beam splitter-like interaction between mechanical degree of freedom and microwave cavity modes. In fact this term allows both electromechanical cooling (with rate $\Gamma_i = 4G_i^2/\kappa_i$) and bidirectional photon conversion between two distinct microwave frequencies. In the photon conversion process, first an input microwave signal at frequency ω_1 with amplitude $a_{\mathrm{in},1}(\omega_1)$ is down-converted into the mechanical mode at frequency ω_m, i.e. $a_1(\omega_1) \xrightarrow{H \propto a_1 b^\dagger} b(\omega_\mathrm{m})$. Next, during an up-conversion process the mechanical mode transfers its energy to the output of the other microwave cavity at frequency ω_2 and amplitude $a_{\mathrm{out},2}(\omega_2)$, i.e. $b(\omega_\mathrm{m}) \xrightarrow{H \propto b a_2^\dagger} a_2(\omega_2)$. Likewise, an input microwave signal at frequency ω_2 can be converted to frequency ω_1 by reversing the conversion process. In fact, the Hermitian aspect of the Hamiltonian (2) makes the conversion process bidirectional and holds the time-reversal symmetry.

The photon conversion efficiency, which is defined as the ratio of the output-signal photon flux over the input-signal photon flux, is given by $|S_{21}|^2 = \left| \frac{a_{\mathrm{out},2(\omega_2)}}{a_{\mathrm{in},1(\omega_1)}} \right|^2$. Since the conversion process is bidirectional therefore $|S_{21}| = |S_{12}| = |T|$. In the steady state and in the weak coupling regime the conversion efficiency reduces to

$$|T|^2 = \frac{4\eta_1\eta_2 C_1 C_2}{(1 + C_1 + C_2)^2}, \tag{3}$$

where $C_{1(2)} = \frac{4g_{0,1(2)}^2 n_{1(2)}}{\kappa_{1(2)}\gamma_\mathrm{m}}$ is the electromechanical cooperativity for cavity 1 (2) and $\eta_{1(2)} = \frac{\kappa_{\mathrm{ext},1(2)}}{\kappa_{1(2)}}$ is the output coupling ratio in which $\kappa_i = \kappa_{\mathrm{int},i} + \kappa_{\mathrm{ext},i}$ is the total damping rate while $\kappa_{\mathrm{int},i}$ and $\kappa_{\mathrm{ext},i}$ show the intrinsic and extrinsic decay rate of the microwave cavities, respectively. Likewise, the reflection coefficients due to impedance mismatch are given by

$$|S_{11}|^2 = \Big(\frac{1 + C_1 + C_2 - 2\eta_1(1 + C_2)}{1 + C_1 + C_2} \Big)^2, \tag{4}$$

$$|S_{22}|^2 = \Big(\frac{1 + C_1 + C_2 - 2\eta_2(1 + C_1)}{1 + C_1 + C_2} \Big)^2. \tag{5}$$

Note that for the lossless microwave cavities ($\eta_i = 1$), near unity photon conversion can be achieved in the limit that $C_1 = C_2 = C$ and $C \gg 1$. The former condition balances the photon-phonon conversion rate for each cavity while the later condition guarantees the mechanical damping rate γ_m is much weaker than the damping rates $\Gamma_i = \gamma_\mathrm{m} C_i$. Under these two conditions, the ideal photon conversion is achieved i.e. $|T|^2 = 1$ (perfect transmission) and $|S_{11}|^2 = |S_{22}|^2 = 0$ (no reflection). The denominator of Eq. (3) indicates that the bandwidth of the conversion is given by $\Gamma_T = \gamma_\mathrm{m} + \Gamma_1 + \Gamma_2$, which is the total back-action-damped linewidth of the mechanical resonator in the presence of the two microwave drive fields.

We perform coherent microwave frequency conversion using the intermediate nanostring resonator as a coupling element between two superconducting coil resonators at $\omega_1/2\pi = 9.55$ GHz and $\omega_2/2\pi = 9.82$ GHz as shown in Fig. 1 a of the main text. The microwave cavities are accessible by ports", i.e. semi-infinite transmission lines giving the modes finite energy decay rates leading to the cavity linewidths $\kappa_1/2\pi = 2.42$ MHz and $\kappa_2/2\pi = 1.98$ MHz with associated output coupling ratios $\eta_1 = 0.74$ and $\eta_2 = 0.86$, indicating that both cavities are strongly overcoupled to the two distinct physical ports 1 and 2. The fundamental mode of the mechanical oscillator has a resonance frequency of $\omega_\mathrm{m}/2\pi = 4.34$MHz with the corresponding damping rate of $\gamma_\mathrm{m}/2\pi = 4$Hz. Measuring the mechanical resonator noise spectrum along with the off-resonant reflection coefficients of each cavity and measurement line, we calibrate the gain and attenuation in each input-output line and accurately back out the vacuum electromechanical coupling rate for each cavity of $g_{01}/2\pi = 33$Hz and $g_{02}/2\pi = 13$Hz.

Supplementary Figure 1 c shows the measured scattering parameters $|S_{11}|^2$ (red line), $|S_{22}|^2$ (blue line), and $|T|^2 = |S_{12}| \cdot |S_{21}|$ (yellow line) versus the electromechanical cooperativity C_2 at $C_1 = 95$. As predicted by Eq. (3) at

$C_1 = C_2 \simeq 95$ we measure a transmission of $|T|^2 = 0.64$, which is dominated by internal losses of the cavities limiting the maximum reachable conversion efficiency to $|T|^2 \leq \eta_1\eta_2 = 0.64$.

Another important aspect of such a transducer is the dynamic range of the device. In the inset of Supplementary Fig. 1 c we show measured maximum transmission as a function of the applied signal power. Our results demonstrate that high conversion efficiencies can be maintained up to about −80 dBm input signal power, corresponding to about 10^5 signal photons inside the cavities. At even higher signal powers the transmission efficiency is degraded abruptly, because the probe tone acts as an additional strong drive invalidating the transducer model, and also because of an increase of the resonance frequency shifts and resonator losses.

Supplementary Note 3: Hamiltonian of a multi-mode electromechanical transducer

In this section we present a general theory to describe the nonreciprocal behaviour of our on-chip electromechanical transducer, shown in Fig 1a of the main paper. We begin with an electromechanical system comprised of three microwave cavities with frequencies ω_i and linewidths κ_i where $i = 1, 2, 3$ that are coupled to two vibrational modes of a mechanical oscillator with frequencies $\omega_{\mathrm{m.i}}$ and damping rates $\gamma_{\mathrm{m},i}$ where $i = 1, 2$. To tune a desired coupling into resonance, we assume the cavities are coherently driven with six microwave tones, with frequencies detuned from the lower motional sidebands of the resonances by $\delta_{0,\mathrm{i}}$. The Hamiltonian of the system is ($\hbar = 1$) [4]

$$H = \sum_{i=1}^{3} \omega_i a_i^\dagger a_i + \sum_{i=1}^{2} \omega_{m,i} b_i^\dagger b_i + \sum_{i=1}^{3}\sum_{j=1}^{2} g_{0,ij} a_i^\dagger a_i (b_j + b_j^\dagger) + H_d, \tag{6}$$

where a_i is the annihilation operator for the cavity i, b_j is the annihilation operator of the mechanical mode j, and

$$H_d = \sum_{i=1}^{3}\sum_{j=1}^{2} \mathcal{E}_{ij} (a_i e^{i(\omega_{\mathrm{d,ij}} t + \phi_{ij})} + a_i^\dagger e^{-i(\omega_{\mathrm{d,ij}} t + \phi_{ij})}), \tag{7}$$

describes the Hamiltonian of the pumps with amplitude $\mathcal{E}_{ij} = \mathcal{E}_{ij}^*$, frequency $\omega_{\mathrm{d,ij}}$, and phase ϕ_{ij}.

We can linearize Hamiltonian (6) by expanding the cavity modes around their steady-state field amplitudes, $a_i \to a_i - \sum_{j=1}^{2} \alpha_{ij} e^{-i\omega_{\mathrm{d,ij}} t}$, where $|\alpha_{ij}|^2 = 4|\mathcal{E}_{ij} e^{-i\phi_{ij}}|^2/(\kappa_i^2 + 4\Delta_{ij}^2)$ is the mean number of photons inside the cavity i induced by the microwave pump due to driving mechanical mode j, the $\kappa_i = \kappa_{\mathrm{int},i} + \kappa_{\mathrm{ext},i}$ is the total damping rate of the cavity while $\kappa_{\mathrm{int},i}$ and $\kappa_{\mathrm{ext},i}$ show the intrinsic and extrinsic decay rate of the microwave cavities, respectively. Here, $\Delta_{ij} = \omega_i - \omega_{\mathrm{d,ij}}$ is the detuning of the drive tone with respect to cavity i. In the rotating frame with respect to $\sum_{i=1}^{3} \omega_i a_i^\dagger a_i + \sum_{i=1}^{2} (\omega_{\mathrm{m},i} + \delta_{0,\mathrm{i}}) b_i^\dagger b_i$, the linearized Hamiltonian becomes

$$H = -\sum_{i=1}^{2} \delta_{0,\mathrm{i}} b_i^\dagger b_i + \sum_{i=1}^{3} \Big\{ \Big(\sum_{j=1}^{2} \big[\alpha_{ij} e^{i\Delta_{ij} t} a_i^\dagger + \alpha_{ij}^* e^{-i\Delta_{ij} t} a_i \big] \Big) \Big(\sum_{j=1}^{2} g_{0,\mathrm{ij}} \big[b_j e^{-i(\omega_{\mathrm{m},j} + \delta_{0,\mathrm{j}})t} + b_j^\dagger e^{i(\omega_{\mathrm{m},j} + \delta_{0,\mathrm{j}})t} \big] \Big) \Big\}. \tag{8}$$

By setting the effective cavity detunings so that $\Delta_{11} = \Delta_{21} = \Delta_{31} = \omega_{\mathrm{m},1} + \delta_{0,1}$ and $\Delta_{12} = \Delta_{22} = \Delta_{32} = \omega_{\mathrm{m},2} + \delta_{0,2}$ and neglecting the terms rotating at $\pm 2\omega_{\mathrm{m},1(2)}$ and $\omega_{\mathrm{m},1} + \omega_{\mathrm{m},2}$, the above Hamiltonian reduces to

$$H = -\sum_{i=1}^{2} \delta_{0,\mathrm{i}} b_i^\dagger b_i + \sum_{i=1}^{3}\sum_{j=1}^{2} \Big(G_{ij} a_i^\dagger b_j + G_{ij}^* a_i b_j^\dagger \Big) + H_{\mathrm{off}}. \tag{9}$$

where $G_{ij} = g_{0,\mathrm{ij}} |\alpha_{ij}| e^{-i\phi_{ij}}$ is the effective coupling rate between the mechanical mode j and cavity i and H_{off} describes off-resonant/time dependent interaction between mechanical modes and the cavity fields, and it is given by

$$H_{\mathrm{off}} = \sum_{i=1}^{3}\sum_{j=1}^{2} \Big[F_{ij} a_i^\dagger b_j e^{(-1)^{j-1} i\delta\omega_{\mathrm{m}} t} + H.c. \Big] \tag{10}$$

where $\delta\omega_{\mathrm{m}} = \omega_{\mathrm{m},2} - \omega_{\mathrm{m},1} + \delta_{0,2} - \delta_{0,1}$ and we define following off-resonant electromechanical coupling parameters

$$\begin{aligned} F_{11} &= g_{0,11} |\alpha_{12}| e^{-i\phi_{12}},\; F_{12} = g_{0,12} |\alpha_{11}| e^{-i\phi_{11}}, \\ F_{21} &= g_{0,21} |\alpha_{22}| e^{-i\phi_{22}},\; F_{22} = g_{0,22} |\alpha_{21}| e^{-i\phi_{21}}, \\ F_{31} &= g_{0,31} |\alpha_{32}| e^{-i\phi_{32}},\; F_{32} = g_{0,32} |\alpha_{31}| e^{-i\phi_{31}}. \end{aligned} \tag{11}$$

The off-resonant Hamiltonian (10) has an essential role in the nonreciprocity aspect of our device, therefore, it is important to discuss the physical roots of such off-resonant couplings [5, 6]. Inspection of Hamiltonians (9) and (10) reveals that each drive tone generates two different types of interactions: Resonant coupling in which the drive tone couples a single mechanical mode to a single cavity mode, described by the time-independent part of the Hamiltonian (9). Each drive tone also generates an interaction which couples the other mechanical mode to the cavity off-resonantly. The Hamiltonians (10) explain this off-resonant coupling between cavity fields and mechanical modes. As we will see, these off-resonant couplings alter the mechanical damping rate, which changes the isolation bandwidth and also cools the mechanical modes. In addition, the coupling also introduces mechanical frequency shifts and introduces an effective detuning for the drive tones. Note that, within the rotating wave approximation (RWA) the non-resonant/time-dependent components of the effective linearized interactions can be neglected in the weak coupling regime and when the cavity decay rates κ_i are much smaller than the two mechanical frequencies $\omega_{\mathrm{m},i}$ and their difference

$$|F_{ij}|, \kappa_i \ll \omega_{\mathrm{m},j}, |\omega_{\mathrm{m},2} - \omega_{\mathrm{m},1}|. \tag{12}$$

Finally, we note that for the isolator case we deal with two cavities coupled two mechanical modes, which mathematically is equivalent to set $G_{31} = G_{32} = F_{31} = F_{32} = 0$ in our general model. In this special case, the Hamiltonian (9) reduces to the Hamiltonian (1) presented in the paper

$$H = -\sum_{i=1}^{2} \delta_{0,\mathrm{i}} b_i^\dagger b_i + \sum_{i,j=1}^{2} \Big(G_{ij} a_i^\dagger b_j + G_{ij}^* a_i b_j^\dagger \Big) + H_{\mathrm{off}}. \tag{13}$$

with

$$H_{\mathrm{off}} = \sum_{i,j=1}^{2} \Big[F_{ij} a_i^\dagger b_j e^{(-1)^{j-1} i\delta\omega_{\mathrm{m}} t} + H.c. \Big]. \tag{14}$$

Supplementary Note 4: Equations of motion and effective model

The full quantum treatment of the system can be given in terms of the quantum Langevin equations where we add to the Heisenberg equations the quantum noise acting on the mechanical resonators $b_{\mathrm{in},i}$ with damping rates γ_i as well as the cavities input fluctuations $a_{\mathrm{in},i}$ with damping rates $\kappa_{\mathrm{ext},i}$. The resulting Langevin equations, including the off-resonant terms, for the cavity modes and mechanical resonators are

$$\dot{a}_i = -\frac{\kappa_i}{2} a_i - i \sum_{j=1}^{2} G_{ij} b_j - i \sum_{j=1}^{2} F_{ij} b_j e^{(-1)^{j-1} i\delta\omega_{\mathrm{m}} t} + \sqrt{\kappa_{\mathrm{ext},i}}\, a_{\mathrm{in},i}. \tag{15}$$

$$\dot{b}_j = \Big(i\delta_{0,\mathrm{j}} - \frac{\gamma_{\mathrm{m},j}}{2} \Big) b_j - i \sum_{i=1}^{3} G_{ij}^* a_i - i \sum_{i=1}^{3} F_{ij}^* a_i e^{(-1)^{j} i\delta\omega_{\mathrm{m}} t} + \sqrt{\gamma_{\mathrm{m},j}}\, b_{\mathrm{in},j}. \tag{16}$$

where $i = 1, 2, 3$ and $j = 1, 2$.

In order to study the dynamics of the system we solve the time-dependent quantum Langevin equations (15). We use an iterative method to solve these equations by defining a new set of auxiliary operators (toy modes) and cutting

the iteration sequence at higher order dependence to $O(n\,\delta\omega_{\rm m};\delta\omega_{\rm m}^n)$ with $n\geq 2$, which yields

$$\dot{a}_i = -\frac{\kappa_i}{2}a_i - i\sum_{j=1}^{2}G_{ij}b_j - i\sum_{j=1}^{2}F_{ij}B_j + \sqrt{\kappa_{{\rm ext},i}}a_{{\rm in},i}, \tag{17}$$

$$\dot{b}_1 = \Big(i\delta_{0,1} - \frac{\gamma_{{\rm m},1}}{2}\Big)b_1 - i\sum_{i=1}^{3}G_{i1}^*a_i - i\sum_{i=1}^{3}F_{i1}^*A_i^- + \sqrt{\gamma_{{\rm m},1}}b_{{\rm in},1}, \tag{18}$$

$$\dot{b}_2 = \Big(i\delta_{0,2} - \frac{\gamma_{{\rm m},2}}{2}\Big)b_2 - i\sum_{i=1}^{3}G_{i2}^*a_i - i\sum_{i=1}^{3}F_{i2}^*A_i^+ + \sqrt{\gamma_{{\rm m},2}}b_{{\rm in},2}, \tag{19}$$

$$\dot{A}_i^+ = (i\delta\omega_{\rm m} - \frac{\kappa_i}{2})A_i^+ - i\Big(F_{i2}b_2 + G_{i1}B_1\Big), \tag{20}$$

$$\dot{A}_i^- = -(i\delta\omega_{\rm m} + \frac{\kappa_i}{2})A_i^- - i\Big(F_{i1}b_1 + G_{i2}B_2\Big), \tag{21}$$

$$\dot{B}_1 = \Big(i[\delta\omega_{\rm m} + \delta_{0,1}] - \frac{\gamma_{{\rm m},1}}{2}\Big)B_1 - i\sum_{i=1}^{3}\Big(F_{i1}^*a_i + G_{i1}^*A_i^+\Big), \tag{22}$$

$$\dot{B}_2 = -\Big(i[\delta\omega_{\rm m} - \delta_{0,2}] + \frac{\gamma_{{\rm m},2}}{2}\Big)B_2 - i\sum_{i=1}^{3}\Big(F_{i2}^*a_i + G_{i2}^*A_i^-\Big). \tag{23}$$

where $i=1,2,3$. The auxiliary modes $A_i^\pm = a_i e^{\pm i\delta\omega_{\rm m}t}$, $B_1 = b_1 e^{i\delta\omega_{\rm m}t}$ and $B_2 = b_2 e^{-i\delta\omega_{\rm m}t}$ describe the off-resonant components of the equations of motion. Here, we take $\delta\omega_{\rm m}$ to be much larger than the relevant system frequencies, i.e. $\delta\omega_{\rm m}\gg\gamma_{{\rm m},i},\delta_{0,{\rm i}},\omega$, and can thus adiabatically eliminate the auxiliary modes by taking $\dot{B}_j = \dot{A}_i^\pm = 0$ in Eqs. (17), which results in the following equations for the auxiliary modes

$$A_i^+ \;=\; \frac{i\Big(F_{i2}b_2 + G_{i1}B_1\Big)}{(i\delta\omega_{\rm m} - \frac{\kappa_i}{2})}, \tag{24}$$

$$A_i^- \;=\; -\frac{i\Big(F_{i1}b_1 + G_{i2}B_2\Big)}{(i\delta\omega_{\rm m} + \frac{\kappa_i}{2})}, \tag{25}$$

$$B_1 \;=\; \frac{i\sum_{i=1}^{3}\Big(F_{i1}^*a_i + G_{i1}^*A_i^+\Big)}{\Big(i[\delta\omega_{\rm m} + \delta_{0,1}] - \frac{\gamma_{{\rm m},1}}{2}\Big)}, \tag{26}$$

$$B_2 \;=\; -\frac{i\sum_{i=1}^{3}\Big(F_{i2}^*a_i + G_{i2}^*A_i^-\Big)}{\Big(i[\delta\omega_{\rm m} - \delta_{0,2}] + \frac{\gamma_{{\rm m},2}}{2}\Big)}. \tag{27}$$

In the limit of $\delta\omega_{\rm m}\to\infty$, the contribution of all auxiliary modes can be totally neglected in the dynamics of the system, i.e. $\{B_j, A_i^\pm\}\to 0$. In this case the off-resonant interactions between the mechanical modes and cavities are negligible and we can safely ignore the time-dependent components of the Hamiltonian (i.e. $H_{\rm off}=0$). However, in our system due to finite value of $\delta\omega_{\rm m}\approx\kappa_i/2$, we cannot ignore these off-resonant interactions.

We can simply further the equations of motion for the main modes by substituting Eqs. (24) into the equations of motion for a_i and b_j in Eqs. (17) and assuming $\delta\omega_{\rm m},\kappa_i\gg\Big\{|\delta_{0,j}|,\gamma_{{\rm m},j},|G_{ij}|,|F_{ij}|\Big\}$,

$$\dot{a}_i \approx -\frac{\kappa_i}{2}a_i - i\sum_{j=1}^{2}G_{ij}b_j + \sqrt{\kappa_{{\rm ext},i}}a_{{\rm in},i}, \tag{28}$$

$$\dot{b}_1 \approx \Big(i\delta_1 - \frac{\Gamma_{{\rm m},1}}{2}\Big)b_1 - i\sum_{i=1}^{3}G_{i1}^*a_i + \sqrt{\gamma_{{\rm m},1}}b_{{\rm in},1}, \tag{29}$$

$$\dot{b}_2 \approx \Big(i\delta_2 - \frac{\Gamma_{{\rm m},2}}{2}\Big)b_2 - i\sum_{i=1}^{3}G_{i2}^*a_i + \sqrt{\gamma_{{\rm m},2}}b_{{\rm in},2}, \tag{30}$$

where $\delta_{\rm j}$ and $\Gamma_{{\rm m},j}$ are the effective detuning and damping rates of the mechanical modes, respectively, and they are

given by

$$\delta_1 = \delta_{0,1} + \delta\omega_{\rm m} \sum_{i=1}^{3} \frac{4|F_{i1}|^2}{4\delta\omega_{\rm m}^2 + \kappa_i^2}, \tag{31}$$

$$\delta_2 = \delta_{0,2} - \delta\omega_{\rm m} \sum_{i=1}^{3} \frac{4|F_{i2}|^2}{4\delta\omega_{\rm m}^2 + \kappa_i^2}, \tag{32}$$

$$\Gamma_{{\rm m},1} = \gamma_{{\rm m},1} + \sum_{i=1}^{3} \frac{4\kappa_i|F_{i1}|^2}{4\delta\omega_{\rm m}^2 + \kappa_i^2}, \tag{33}$$

$$\Gamma_{{\rm m},2} = \gamma_{{\rm m},2} + \sum_{i=1}^{3} \frac{4\kappa_i|F_{i2}|^2}{4\delta\omega_{\rm m}^2 + \kappa_i^2}. \tag{34}$$

Note that in the derivation of Eqs. (28) we assume that the off-resonant interaction does not considerably modify the self-interaction and damping rate of the cavity modes. Inspection of Eqs. (28) reveals that the off-resonant coupling between mechanical modes and cavities shifts the resonance frequency and damps/cools the mechanical modes by introducing a cross-damping between them. The strength of the frequency shift and the cross-damping is given by the off-resonant electromechanical coupling parameters F_{ij}, which indicates that the drive tones creates an effective coupling between the two mechanical modes. In the weak coupling regime and for very large $\delta\omega_{\rm m}$ this cross-coupling is negligible, thus $\delta_{\rm j} \approx \delta_{0,{\rm j}}$ and $\Gamma_{{\rm m},j} \approx \gamma_{{\rm m},j}$.

We can solve the Eqs. (28) in the Fourier domain to obtain the microwave cavities' variables. Eliminating the mechanical degrees of freedom from the equations of motion (28) and writing the remaining equations in the matrix form, we obtain

$$\Big(\mathbf{M} - i\omega\mathbf{I}\Big) \begin{pmatrix} a_1 \\ a_2 \\ a_3 \end{pmatrix} = \begin{pmatrix} \sqrt{\kappa_{{\rm ext},1}}a_{{\rm in},1} - iG_{11}\chi_{{\rm m},1}(\omega)\sqrt{\gamma_{{\rm m},1}}b_{{\rm in},1} - iG_{12}\chi_{{\rm m},2}(\omega)\sqrt{\gamma_{{\rm m},2}}b_{{\rm in},2} \\ \sqrt{\kappa_{{\rm ext},2}}a_{{\rm in},2} - iG_{21}\chi_{{\rm m},1}(\omega)\sqrt{\gamma_{{\rm m},1}}b_{{\rm in},1} - iG_{22}\chi_{{\rm m},2}(\omega)\sqrt{\gamma_{{\rm m},2}}b_{{\rm in},2} \\ \sqrt{\kappa_{{\rm ext},3}}a_{{\rm in},3} - iG_{31}\chi_{{\rm m},1}(\omega)\sqrt{\gamma_{{\rm m},1}}b_{{\rm in},1} - iG_{32}\chi_{{\rm m},2}(\omega)\sqrt{\gamma_{{\rm m},2}}b_{{\rm in},2} \end{pmatrix}, \tag{35}$$

where $\chi_j^{-1}(\omega) = \Gamma_{{\rm m},j}/2 - i(\omega + \delta_{\rm j})$ is the mechanical susceptibility for mode j and we introduced the drift matrix

$$\mathbf{M} = \begin{pmatrix} \frac{\kappa_1}{2} + \chi_{{\rm m},1}(\omega)|G_{11}|^2 + \chi_{{\rm m},2}(\omega)|G_{12}|^2 & \chi_{{\rm m},1}(\omega)G_{11}G_{21}^* + \chi_{{\rm m},2}(\omega)G_{12}G_{22}^* & \chi_{{\rm m},1}(\omega)G_{11}G_{31}^* + \chi_{{\rm m},2}(\omega)G_{12}G_{32}^* \\ \chi_{{\rm m},1}(\omega)G_{11}^*G_{21} + \chi_{{\rm m},2}(\omega)G_{12}^*G_{22} & \frac{\kappa_2}{2} + \chi_{{\rm m},1}(\omega)|G_{21}|^2 + \chi_{{\rm m},2}(\omega)|G_{22}|^2 & \chi_{{\rm m},1}(\omega)G_{31}^*G_{21} + \chi_{{\rm m},2}(\omega)G_{32}^*G_{22} \\ \chi_{{\rm m},1}(\omega)G_{11}^*G_{31} + \chi_{{\rm m},2}(\omega)G_{12}^*G_{32} & \chi_{{\rm m},1}(\omega)G_{21}^*G_{31} + \chi_{{\rm m},2}(\omega)G_{22}^*G_{32} & \frac{\kappa_3}{2} + \chi_{{\rm m},1}(\omega)|G_{31}|^2 + \chi_{{\rm m},2}(\omega)|G_{32}|^2 \end{pmatrix}.$$

By substituting the solutions of Eq. (35) into the corresponding input-output formula for the cavities variables, i.e. $a_{{\rm out},j} = \sqrt{\kappa_{{\rm ext},j}}a_j - a_{{\rm in},j}$, we obtain

$$\begin{pmatrix} a_{{\rm out},1} \\ a_{{\rm out},2} \\ a_{{\rm out},3} \end{pmatrix} = \mathbf{T}.\Big(\mathbf{M} - i\omega\mathbf{I}\Big)^{-1}. \begin{pmatrix} \sqrt{\kappa_{{\rm ext},1}}a_{{\rm in},1} - iG_{11}\chi_{{\rm m},1}(\omega)\sqrt{\gamma_{{\rm m},1}}b_{{\rm in},1} - iG_{12}\chi_{{\rm m},2}(\omega)\sqrt{\gamma_{{\rm m},2}}b_{{\rm in},2} \\ \sqrt{\kappa_{{\rm ext},2}}a_{{\rm in},2} - iG_{21}\chi_{{\rm m},1}(\omega)\sqrt{\gamma_{{\rm m},1}}b_{{\rm in},1} - iG_{22}\chi_{{\rm m},2}(\omega)\sqrt{\gamma_{{\rm m},2}}b_{{\rm in},2} \\ \sqrt{\kappa_{{\rm ext},3}}a_{in,3} - iG_{31}\chi_{{\rm m},1}(\omega)\sqrt{\gamma_{{\rm m},1}}b_{{\rm in},1} - iG_{32}\chi_{{\rm m},2}(\omega)\sqrt{\gamma_{{\rm m},2}}b_{{\rm in},2} \end{pmatrix} - \begin{pmatrix} a_{{\rm in},1} \\ a_{{\rm in},2} \\ a_{{\rm in},3} \end{pmatrix}, \tag{36}$$

where we defined $\mathbf{T} = {\rm Diag}\big[\sqrt{\kappa_{{\rm ext},1}}, \sqrt{\kappa_{{\rm ext},2}}, \sqrt{\kappa_{{\rm ext},3}}\big]$.

Supplementary Note 5: Scattering matrix and nonreciprocity for a two-port device

In this section, we verify the details of our analysis in the isolator section of the main paper and we examine our model to see how the nonreciprocity arises in a two-port electromechanical system. Here, we are only interested in the response an electromechanical system comprised of two microwave cavities and two mechanical modes. Therefore, by setting $G_{3{\rm j}} \to 0$ and $\delta_1 = -\delta_2 = \delta$ in Eq. (36) and assuming $\phi_{22} = \phi$, $\phi_{11} = \phi_{12} = \phi_{21} = 0$, we can find the ratio of backward to forward transmission

$$\lambda := \frac{S_{12}(\omega)}{S_{21}(\omega)} = \frac{\sqrt{C_{11}C_{21}}\Sigma_{{\rm m},2}(\omega) + \sqrt{C_{12}C_{22}}\Sigma_{{\rm m},1}(\omega)e^{i\phi}}{\sqrt{C_{11}C_{21}}\Sigma_{{\rm m},2}(\omega) + \sqrt{C_{12}C_{22}}\Sigma_{{\rm m},1}(\omega)e^{-i\phi}}, \tag{37}$$

as specified in Eq. (2) of the paper. Here, $\Sigma_{{\rm m},j} = 1 + 2i\big[(-1)^j\delta - \omega\big]/\Gamma_{{\rm m},j}$ is the inverse of the mechanical susceptibility divided by the effective mechanical linewidth $\Gamma_{{\rm m},j}$. Examination of Eq. (37) shows that the nominator and

denominator of this equation are not equal and they possess different relative phase. This asymmetry is the main source of the nonreciprocity and appearance of isolation in the system. In particular, at a phase

$$e^{i\phi} = -\sqrt{\frac{C_{11}C_{21}}{C_{12}C_{22}}\frac{\Sigma_{\mathrm{m},2}(\omega)}{\Sigma_{\mathrm{m},1}(\omega)}}, \tag{38}$$

the nominator of the Eq. (37) will be zero, therefore, backward transmission S_{12} is cancelled while forward transmission S_{21} is non-zero. Rewriting Eq. (38) gives

$$\tan[\phi(\omega)] = \frac{\delta(\Gamma_{\mathrm{m},1}+\Gamma_{\mathrm{m},2}) + \omega(\Gamma_{\mathrm{m},2}-\Gamma_{\mathrm{m},1})}{\Gamma_{\mathrm{m},1}\Gamma_{\mathrm{m},2}/2 - 2(\delta^2-\omega^2)}. \tag{39}$$

By neglecting the contribution of the off-resonant term in the response of the system, i.e. $\Gamma_{\mathrm{m},j} \to \gamma_{\mathrm{m},j}$ the Eq. (39) reduces to Eq. (3) of the paper. At the optimum phase (38) and at cavity resonance, the transmission in the forward direction is given by

$$S_{21} = -\frac{2\sqrt{\eta_1\eta_2}\left[\Sigma_{\mathrm{m},1}(0)\Sigma_{\mathrm{m},2}(0)\right]\left(\sqrt{C_{11}C_{21}}\Sigma_{\mathrm{m},2}(0) + \sqrt{C_{12}C_{22}}\Sigma_{\mathrm{m},1}(0)e^{-i\phi}\right)}{\left[C_{11}\Sigma_{\mathrm{m},2}(0) + C_{12}\Sigma_{\mathrm{m},1}(0) + \Sigma_{\mathrm{m},1}(0)\Sigma_{\mathrm{m},2}(0)\right]\left[C_{21}\Sigma_{\mathrm{m},2}(0) + C_{22}\Sigma_{\mathrm{m},1}(0) + \Sigma_{\mathrm{m},1}(0)\Sigma_{\mathrm{m},2}(0)\right]}.$$

For equal mechanical damping $\Gamma_{\mathrm{m},1} = \Gamma_{\mathrm{m},2} = \Gamma$ (equivalent to $\gamma_{\mathrm{m},1} = \gamma_{\mathrm{m},2} = \gamma$ of the main text) and at equal cooperativities for all four electromechanical couplings ($C_{ij} = \mathcal{C}$) the above equation reduces to

$$S_{21} = -\sqrt{\eta_1\eta_2}\Big[\frac{4i\,\delta(1-2i\delta/\Gamma)}{\mathcal{C}\Gamma(1+\frac{1+4\delta^2/\Gamma^2}{2\mathcal{C}})^2}\Big] \tag{40}$$

as specified in Eq. (4) of the paper. For the particular cooperativity $2\mathcal{C} = 1 + 4\delta^2/\Gamma^2$, the power transmission in forward direction is given by

$$|S_{21}|^2 = \eta_1\eta_2\Big(1 - \frac{1}{2\mathcal{C}}\Big). \tag{41}$$

By neglecting the off-resonant interaction all damping rates reduce to $\Gamma_{\mathrm{m},j} \approx \gamma_{\mathrm{m},j}$ which is consistent with our notation in the main text. We also note that the frequency shifts due to off-resonant interaction for the isolator system discussed in the main text are given by $(\delta_1, \delta_2)/2\pi = (-84, 233)$ Hz while the cross-damping rates are $(\Gamma_{\mathrm{m},1}, \Gamma_{\mathrm{m},2})/2\pi = (190, 407)$ Hz.

Supplementary Note 6: Theoretical model for the circulator

The theoretical model, we presented in Eqs. (17), or equivalently Eq. (36), fully describes the nonreciprocal behaviour of the system for the case of the circulator. In order to check this, in Supplementary Fig. 2 we show both measured experimental data and the theoretical prediction. The theoretical model is in excellent agreement with the experiment and can perfectly describe the nonreciprocity of photon transmission for both forward and backward circulation. The frequency shifts due to off-resonant interaction for the circulator system discussed in the main text are given by $(\delta_1, \delta_2)/2\pi = (-90.6, 329)$ Hz and the cross-damping rates are $(\Gamma_{\mathrm{m},1}, \Gamma_{\mathrm{m},2})/2\pi = (209.9, 624.9)$ Hz.

Supplementary Note 7: Scattering parameters and noise properties of the circulator

In this section, we discuss the noise properties of the system and present data for the added noise during the frequency conversion when operated as a circulator.

Equation (36) explains that due to the linear nature of the input-output theorem and in the absence of the input coherent signal, the output of each cavity is a linear combination of the electromagnetic input noise $a_{\mathrm{in},i}$ and mechanical noise $b_{\mathrm{in},j}$. Therefore, Eq. (36) can be rewritten in the following general form

$$a_{\mathrm{out},i} = \sum_{j=1}^{3} S_{i,j} a_{\mathrm{in},j} + \sum_{j=1}^{2} T_{i,j} b_{\mathrm{in},j}, \tag{42}$$

where $S_{i,j}$ and $T_{i,j}$ are the scattering matrices. Operating under the white noise assumption, the zero-mean quantum fluctuations $a_{\mathrm{in},i}$ and $b_{\mathrm{in},j}$ satisfy the correlations $\langle O_{\mathrm{in},i/j}(t) O^{\dagger}_{\mathrm{in},i/j}(t')\rangle = (\bar{N}_{i/j}+1)\delta(t-t')$, $\langle O^{\dagger}_{\mathrm{in},i/j}(t) O_{\mathrm{in},i/j}(t')\rangle = \bar{N}_{i/j}\delta(t-t')$, and $\langle O_{\mathrm{in},i/j}(t) O_{\mathrm{in},i/j}(t')\rangle = 0$ where $i = 1,2,3$ for $O = a$, and $j = 1,2$ for $O = b$) and $\bar{N}_{i/j} = 1/\big\{\exp\big[\hbar\omega_i/(k_{\mathrm{B}}T_i)\big]-1\big\}$ ($\bar{N}_{\mathrm{m},j} = 1/\big\{\exp\big[\hbar\omega_{\mathrm{m},j}/(k_{\mathrm{B}}T_j)\big]-1\big\}$) are the thermal photon (phonon) occupancies of the cavities (mechanical resonator) for $i = 1,2,3$ ($j = 1,2$) at temperature T_i. The output of the cavities are then sent through a chain of amplifiers. The electromagnetic modes at the output of the amplifiers are given by

$$A_{\mathrm{out},i} = \big(\sqrt{G_i} a_{\mathrm{out},i} + \sqrt{G_i - 1} c^{\dagger}_{\mathrm{amp},i}\big), \tag{43}$$

where G_i is the effective gain of the amplifier chain at port i and $c_{\mathrm{amp},i}$ is the added noise operator of the amplifiers. We can now write the expression for the single sided power spectral density as measured by a spectrum analyzer, in the presence of all relevant noise sources

$$S_{\mathrm{noise},i}(\omega) = \hbar\omega \int_{-\infty}^{\infty} d\omega' \langle A^{\dagger}_{\mathrm{out},i}(\omega') A_{\mathrm{out},i}(\omega')\rangle. \tag{44}$$

Substituting Eqs. (42) and (43) into Eq. (44), assuming $G_i \approx G_i - 1 = 10^{\mathcal{G}_i/10}$ where $\mathcal{G}_i$ is the gain in dB, and using the white correlation functions for the noise operators, we find

$$S_{\mathrm{noise},i}(\omega) = \hbar\omega 10^{\mathcal{G}_i/10}(1 + n_{\mathrm{amp},i} + n_{\mathrm{add},ij}), \tag{45}$$

where $n_{\mathrm{amp},ij}$ is the total noise added by the amplifier chains and $n_{\mathrm{add},i}$ is the total noise added by the cavities and mechanical resonators associated with the photon conversion from cavity j to cavity i.

Measuring the output noise spectrum and having calibrated the gain of the amplifiers at each port $(\mathcal{G}_1, \mathcal{G}_2, \mathcal{G}_3) = (67.5, 64, 60.5)$ dB, we can accurately infer the amplifiers added noise quanta at each port $(n_{\mathrm{amp},1}, n_{\mathrm{amp},2}, n_{\mathrm{amp},3}) = (23, 23, 33) \pm 2$. The only remaining unknown parameter in Eq. (45) is $n_{\mathrm{add},ij}$ which can be found by measuring the noise properties of the three cavities when all six pumps are on and compare them to the case when the pumps are off. In Supplementary Fig. 3 we show the measured added noise photons for all six transmission parameters of the circulator. Assuming that the signal power is chosen well below the pump power it includes all unwanted noises, potential spurious modes, RF leakage and phase noise from the pumps over the relevant bandwidth. On resonance where the directionality is maximized we find $(n_{\mathrm{add},21}, n_{\mathrm{add},32}, n_{\mathrm{add},13}) = (4, 6.5, 3.6)$ in the forward direction and $(n_{\mathrm{add},12}, n_{\mathrm{add},23}, n_{\mathrm{add},31}) = (4, 4, 5.5)$ in the backward direction.

SUPPLEMENTARY REFERENCES

[1] Pitanti, A. *et al.* Strong opto-electro-mechanical coupling in a silicon photonic crystal cavity. *Opt. Express* **23**, 3196–3208 (2015).

[2] Fink, J. M. *et al.* Quantum electromechanics on silicon nitride nanomembranes. *Nature Communications* **7**, 12396– (2016).

[3] Regal, C. A., Teufel, J. D. & Lehnert, K. W. Measuring nanomechanical motion with a microwave cavity interferometer. *Nat. Phys.* **4**, 555–560 (2008).

[4] Barzanjeh, S. *et al.* Microwave Quantum Illumination. *Phys. Rev. Lett.* **114**, 080503 (2015).

[5] Noguchi, A. *et al.* Strong coupling in multimode quantum electromechanics. *https://arxiv.org/abs/1602.01554* (2016).

[6] Buchmann, L. F. & Stamper-Kurn, D. M. Nondegenerate multimode optomechanics. *Phys. Rev. A* **92**, 013851 (2015).
